# Effect of Oxygen Variation on High Cycle Fatigue Behavior of Ti-6Al-4V Titanium Alloy

**DOI:** 10.3390/ma13173858

**Published:** 2020-09-01

**Authors:** Luyao Tang, Jiangkun Fan, Hongchao Kou, Bin Tang, Jinshan Li

**Affiliations:** 1 State Key Laboratory of Solidification Processing, Northwestern Polytechnical University, Xi’an 710072, China; christinatang@mail.nwpu.edu.cn (L.T.); hchkou@nwpu.edu.cn (H.K.); toby@nwpu.edu.cn (B.T.); 2 National & Local Joint Engineering Research Center for Precision Thermoforming Technology of Advanced Metal Materials, Xi’an 710072, China

**Keywords:** Ti-6Al-4V, oxygen, microstructural characteristic, high cycle fatigue behavior, failure mechanism

## Abstract

The element oxygen is expected to be a low-cost, strengthening element of titanium alloys due to its strong solid solution strengthening effect. High cycle fatigue behaviors of Ti-6Al-4V alloys with different oxygen contents (0.17%, 0.20%, 0.23% wt.%) were investigated in this paper. The results illustrated that Ti-6Al-4V-0.20O alloy possesses the highest fatigue strength and the lowest fatigue crack propagation rate. The fatigue fracture morphology verified that the fatigue cracks propagated transgranularly in both Ti-6Al-4V-0.17O and Ti-6Al-4V-0.20O alloys, and the fatigue cracks tended to extend intergranularly in the Ti-6Al-4V-0.23O alloy. The maximum nano-hardness varied from the <0001> direction to the $<\bar{1}2\bar{1}0>$ and $<01\bar{1}0>$ directions with the increasing oxygen content, which suggested that the dominant slip system varied from prismatic slip to pyramidal slip. The number of the $<\vec{c}+\vec{a}>$ type dislocations increased with the oxygen content, which indicated that the number of the first-order pyramidal and the second-order pyramidal $<\vec{c}+\vec{a}>$ slip systems increased. The oxygen can significantly change the fatigue fracture mechanism of Ti-6Al-4V alloy: From transgranular fracture to intergranular fracture. These results are expected to provide valuable reference for the optimization of the composition and mechanical properties of titanium alloys.

## 1. Introduction

Ti-6Al-4V alloy can maintain excellent long-term comprehensive mechanical properties under 400 °C. Thus, it has been applied extensively on key components such as turbine disks, compressor disks, aero-engine fan blades, etc. [1,2]. There are many inducements that will lead to the failures of the aforementioned key components. Statistically, the failures of turbine engines, which are generated by fatigue, (including high cycle fatigue (HCF) and low cycle fatigue (LCF)), account for over 30% of the total failure accidents, among which the number of HCF failures takes up the overwhelming majority [3,4].

The element oxygen is one of the alpha phase stabilizer elements of titanium alloys [5,6]. Hence, the microstructural characteristics [6,7,8,9,10,11,12] and the mechanical properties [13,14,15] will be strongly influenced by oxygen addition. Welsch et al. [8] found that the volume fraction of alpha phase in Ti-6Al-4V alloy increased from 78.5% to 85.2% with the oxygen content increased from 0.07% to 0.19% by weight percent. Some research conclusions indicate that the average size of alpha grains diminishes with the increasing oxygen content [7,9], which can be ascribed to that oxygen tended to segregate at grain boundaries and dislocations, and hinder dislocation glide/creep and grain boundary migration [10]. The oxygen atom prefers occupying the octahedral interstices of the alpha-Ti lattice, which leads to the expansion of lattice and the lowest formation energy of Ti–O bonding [11,12]. Previous research revealed that the addition of oxygen in titanium alloys can enhance the strength, the elasticity modulus, and the hardness and significantly reduce the ductility of titanium alloys [13,14,15].

In addition, many investigations have been conducted to study the effect of oxygen variation on fatigue properties of titanium alloys, but the experimental results and theoretical analysis are still controversial. Yoder et al. [16] found that the variation of the oxygen content in the Ti-6Al-4V alloys had strong influence on the size of the primary beta grain and the Widmanstätten packet, and then the fatigue crack propagation rate would be significantly affected. However, the microstructure of the beta-annealed Ti-6Al-4V alloys with various oxygen contents varied greatly, and the Widmanstätten sizes ranged from 17–38 μm, while the primary beta grain sizes covered the range of 214–844 μm. Consequently, the authors did not come up with the accurate relationship between the oxygen content and the fatigue crack propagation rate. Not only the oxygen variation of the titanium alloy has remarkable effect on the fatigue crack propagation rate, but also the testing atmosphere influences the rate significantly [17,18]. Hornberger et al. [17] discovered that the titanium alloy would react with the oxygen in the air, which would lead to the formation of the oxide at the specimen surface. Stress concentration will form on the surface oxides and further form fatigue cracks, eventually leading to a significant reduction in fatigue life. Bache and co-workers [19] surveyed the fatigue crack propagation rate of the Ti-6Al-4V alloy in vacuum, argon, and atmosphere, respectively, and they obtained a conclusion consistent with the above. Additionally, they found that the fatigue crack propagation rate sped up significantly with the increasing oxygen content in the Ti-6Al-4V alloy. However, there were many objections on this. For example, Robinson [20] declared that the fatigue crack propagation rate in pure titanium decreased with the increasing oxygen content. The factors, such as alloying element, microstructure features, manufacturing process, etc., have strong influence on the fatigue properties of the titanium alloys, leading to the contradictory conclusions of existing studies. However, there has not been a conclusive assertion pertaining to the issue of the effect of the oxygen content on the fatigue properties of titanium alloys. So, it is essential to study the effect of oxygen variation on the microstructural characteristic and high cycle fatigue behavior of Ti-6Al-4V alloy.

Based on the issues above, Ti-6Al-4V alloys with different oxygen contents (0.17 wt.%, 0.20 wt.%, and 0.23 wt.%, respectively) were investigated in this paper using optical microscope (OM), X-ray diffraction (XRD), scanning electron microscope (SEM), electron back-scattered diffraction (EBSD), transmission electron microscope (TEM), nano-indentation tests, and HCF tests to acquire the accurate effect of oxygen variation on the microstructural characteristics and the fatigue behaviors of Ti-6Al-4V alloy.

## 2. Materials and Experimental Procedure

### 2.1. Materials

The Ti-6Al-4V-*x*O alloys used in this paper were obtained through vacuum arc melting and forging. By controlling the addition of titanium oxide (TiO_2_) during the melting process, Ti-6Al-4V alloys with different oxygen contents can be obtained. More precisely, considering that there may be a 0.03% increase in oxygen content during the whole processing, the weight fraction of TiO_2_ added to the melt during vacuum arc melting process was 0.238%, 0.358%, and 0.433%, respectively, to obtain Ti-6Al-4V alloy with different oxygen contents. The chemical compositions by weight percent of the present Ti-6Al-4V alloys are shown in Table 1, and the oxygen contents of these three kinds of Ti-6Al-4V alloys are 0.17 wt.%, 0.20 wt.%, and 0.23 wt.%, respectively. The beta transus temperatures (*T_β_*) of each alloy were 1000–1005 °C, 1005–1010 °C, and 1015–1020 °C, respectively, which accords with the rule that there will be a 5–10 °C increment of the *T_β_* for every 0.03% increment of oxygen content by weight for titanium alloys [18].

### 2.2. Microstructural Characteristics

OM was employed to obtain the micrographs, which revealed the microstructural characteristics of the Ti-6Al-4V alloys. The process of sample preparation included grinding with metallographic abrasive papers and electrolytic polishing at 20 °C with HClO_4_:C_2_H_5_OH = 1:4 (volume ratio) electrolyte, 30 V electrolytic voltage, and 40 s duration. Then the OM micrographs were analyzed by the software *Image-Pro Plus 6.0* (MEDIA CYBERNETICS, Rockville, MD, USA),using the contrast of the alpha and beta phases, to obtain the microstructural characteristics, such as volume fraction, alpha phase grain size, etc., of the Ti-6Al-4V alloys. XRD was employed to obtain the spectra, which revealed the crystallographic characteristics of the Ti-6Al-4V alloys. The XRD spectra were analyzed by the software *MDI JADE 5.0* (Materials Data (MDI), Livermore, CA, USA).

### 2.3. High Cycle Fatigue Preformance Test

**Figure 1:**
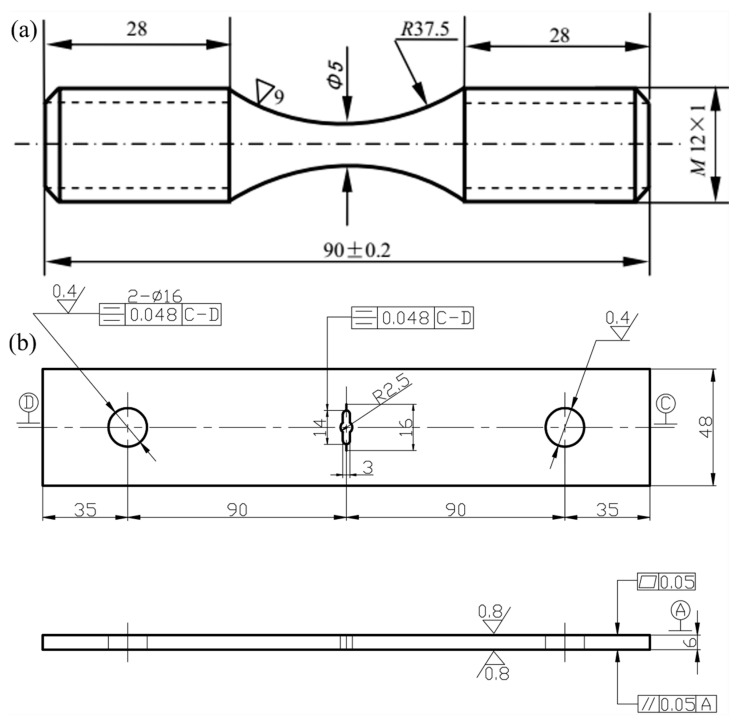
Schematic diagrams of the specimens (unit mm) for (**a**) High Cycle Fatigue (HCF) test and (**b**) fatigue crack growth rate test.

The high cycle fatigue test was conducted on the QBG-100 High Frequency Fatigue Tester (Changchun Qianbang Testing Equipment Co., Ltd., Jilin, China) according to the Chinese national standard GB/T 3075-2008 metallic materials, fatigue testing, axial force-controlled method. The central section of the smooth specimen (*K_t_* = 1, *K_t_* refers to the theoretical stress concentration factor) employed in high cycle fatigue test was a large circular arc, which was polished carefully before tests, as illustrated in Figure 1a. The experimental conditions were as follows: Stress ratio *R* was −1, frequency *f* was 130–140 Hz, and testing temperature *T* was 25 °C.

The calculational method, which was the so-called “staircase” method, of the fatigue limit, which is given in the Chinese national standard GB/T 3075-2008 metallic materials, fatigue testing, axial force-controlled method, was employed to roughly estimate the fatigue strength of the Ti-6Al-4V alloys with different oxygen contents. Such method stipulates that if the former sample damages while the fatigue loading cycle is less than 10^7^, which corresponds to so-called “run out”, the later sample will be tested at a lower stress level. Nevertheless, if the former sample survives, the later test will be conducted at a higher stress level and the above procedures will be repeated until the end of the experiment. The fatigue strength depends on the specified survival rate and fiducial probability. In this paper, the specified survival rate is 50% and the specified fiducial probability is 95%. The calculational equations of the fatigue limit $\hat{\mu }$ and the standard deviation $\hat{\delta }$ are shown in the equations below.

(1) $$\hat{\mu }=S_{0}+d\;(\frac{A}{C}+\frac{1}{2})$$

(2) $$\hat{\delta }=1.62d\;(D+0.029)$$

*S*_0_ is the minimum stress level that the samples survive and *d* is the stress step, the differential between adjacent two stress levels. *A* = Σ*if_i_*, *B* = Σ*i*^2^*f_i_*, *C* = Σ*f_i_, D* = (*BC − A*^2^)/*C*^2^, *i* is the sequence number of the stress applied during tests, and *f_i_* is the number of the samples that survived under different stress levels.

The schematic diagram of the specimens used for fatigue crack propagation rate test is shown in Figure 1b. It can be seen from the figure that the sample was precracked, and the precrack length a is 1 mm. The experimental conditions were constructed on the basis of the Chinese national standard GB/T 6398-2000 Standard Test Method for Fatigue Crack Growth Rates of Metallic Materials. The stress ratio *R* was determined to be −1 and the frequency *f* was chosen to be 50 Hz, while the testing temperature *T* was 25 °C. During the whole experimental process, the crack tip was monitored. The fatigue loading cycle and the length of the fatigue crack were recorded manually. Then, the software *MATLAB 7.10* R2010a was employed to perform data conversion, videlicet, convert the cycles and the lengths of the cracks into the fatigue crack propagation rate (*da/dN*) and the variation range of stress intensity factor (Δ*K*), where *a* represents the length of fatigue crack and *N* is the fatigue loading cycle. The fatigue crack propagation rate obtained by this way accorded well with the II region of the Paris Formula.

### 2.4. Fracture Characteristics Observation and Nano-Indentation Test

In this paper, HCF fracture characteristics include the following parts: Fatigue fracture morphology, fatigue crack propagation path, and the characteristic of the dislocations introduced into Ti-6Al-4V alloy by cyclic loading. SEM was employed to observe the fatigue fractures of Ti-6Al-4V alloys with different oxygen contents. Before the SEM observation, the fatigue fractures were carefully cleaned by ultrasonic cleaning technology to ensure the fractures were clean. In order to study the effect of oxygen variation on the HCF behavior of Ti-6Al-4V alloy, EBSD was used to determine the fatigue crack propagation path in the Ti-6Al-4V alloy. The sample preparation method was different from the above EBSD sample preparation method. The samples employed for fatigue crack propagation path observation were first ground by metallographic abrasive papers and then electrolytically polished at room temperature. The electrolyte was HClO_4_:C_2_H_5_OH = 1:4 (volume ratio), the electrolytic voltage was 30 V, and the duration was 40 s. During the fatigue loading process, a large number of dislocations were introduced into the Ti-6Al-4V alloy. In order to study the effect of oxygen content on the dislocation characteristics, the samples were observed by TEM. Sheets with a thickness of 0.3 mm were cut from the vicinity of the fatigue fracture by wire cutting, and the sheets were ground to 50 to 70 μm with sandpaper. Then, the sheets were subjected to twin-jet electro-polishing in order to obtain a thin zone suitable for TEM characterization. The electrolyte was C_2_H_5_OH:HClO_4_ = 95:5 (volume ratio), using a 30 V electrolytic voltage for 80 s at a temperature below −20 °C during electro-polishing.

The crystal structure of the alpha phase was close-packed hexagonal (HCP), and it was strongly anisotropic, so that the micro-mechanical property of the alpha phase was strongly orientation dependent. Gong et al. [19] proved, by micro-cantilever test, that the critical resolved shear stress (CRSS) required to activate the $<\vec{c}+\vec{a}>$ type dislocations ($11\bar{2}3$) was nearly 3.5 times above that of the $<\vec{a}>$ type ($11\bar{2}0$) dislocations, which was due to the orientation dependency. Thus, the nano-indentation test and the EBSD test were combined to exclude the influence of grain orientation and obtain the effect of oxygen variation on the nano-indentation hardness (*H*) and the elasticity modulus (*E*) of the Ti-6Al-4V alloy.

Nano-indentation test was conducted on the Hysitron In Situ Picoindenter and the Berkovich indenter was employed. The loading process was as follows: The load reached the preset maximum value after loading for 5 s, then the load was kept for 2 s, and finally the unloading process lasted 5 s. The loading curve and unloading curve obtained from the nano-indentation test showed that the sample first elastically deformed during the loading process, and then significant discontinuous feature can be observed through the loading curve, indicating that the Ti-6Al-4V alloy entered the plastic deformation stage. Similarly, during the unloading process, the Ti-6Al-4V alloy first elastically deformed, so the upper section of the unloading curve was basically linear. After the elastic recovery disappeared, the indenter continued to rise away from the surface of the material, but the unloading curve could not return to the origin due to plastic deformation [20]. By using the power function to fit the unloading curve [21] during the indentation test, the nano-indentation hardness (*H*) and the elasticity modulus (*E*) of the Ti-6Al-4V alloys could be calculated. EBSD was applied to samples that were subjected to nano-indentation tests, to determine the orientation of the indented grains and further clarify the influence of grain orientation on micro-mechanical properties of the alpha phase. The EBSD test was carried out on the TESCAN MIRA3 field emission scanning electron microscope equipped with an electron back-scattered diffraction probe, which is produced by TESCAN ORSAY HOLDING, a.s., Brno, Czech Republic. The operating voltage was 20 kV and the step size was 0.18 μm during data acquisition. After data acquisition, the software *HKL Channel 5* copyrighted by the Oxford Instrument Corporation, Abingdon, UK was employed to obtain the orientation of the indented grain. The hardness inverse pole figures (HIPF) and elasticity modulus inverse pole figures (MIPF) were obtained by combining the above grain orientation, the nano-indentation hardness, and the elasticity modulus. Both the samples used for nano-indentation test and for EBSD test were required to be residual stress-free. So, after grinding with metallographic abrasive papers, the vibratory polishing for up to 10 h was employed to diminish the surface residual stress with 0.05 μm SiO_2_ solution.

## 3. Results and Discussion

### 3.1. Microstructural Characteristics of Ti-6Al-4V Alloy

**Figure 2:**
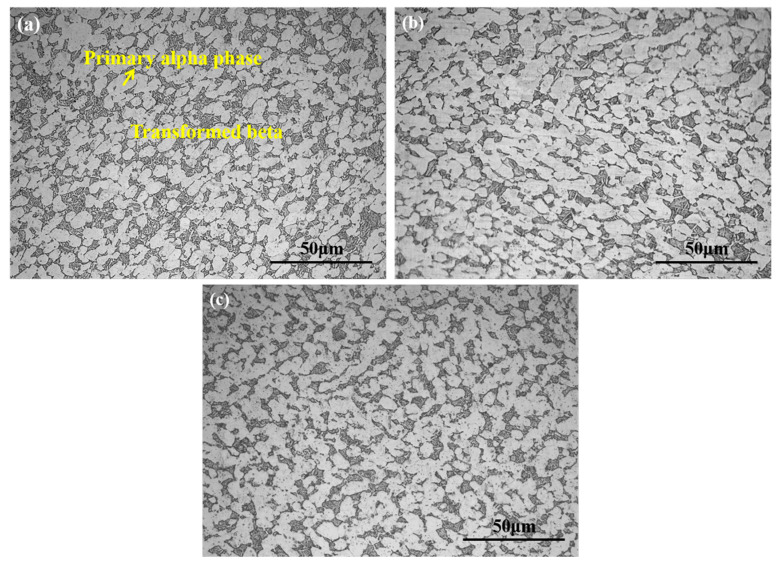
Microstructural characteristics of Ti-6Al-4V alloy (**a**) Ti-6Al-4V-0.17O, (**b**) Ti-6Al-4V-0.20O, (**c**) Ti-6Al-4V-0.23O.

Microstructural characteristics the Ti-6Al-4V alloys with different oxygen contents are demonstrated in Figure 2. The microstructures consisted of coarser equiaxed alpha phase and transformed beta (acicular α + β). In Ti-6Al-4V-0.17O alloy, the volume fraction of the primary equiaxed α phase was approximately 78.3% ± 1.78%. With further increase of oxygen content, the volume fraction of the primary equiaxed α phase in Ti-6Al-4V alloy gradually increased to 81.4 ± 1.23% and 82.9 ± 1.71%. Consequently, the alpha phase volume fractions in three kinds of Ti-6Al-4V alloys were 80% approximately, and increased with the increasing oxygen content in Ti-6Al-4V alloy. Because the element oxygen is one of the most effective alpha phase stabilizer elements of titanium alloys, the *T*_β_ will be raised with the increasing oxygen content. As a consequence, after the same process, there will be more primary alpha phase in the Ti-6Al-4V alloy with higher oxygen content. However, the oxygen increment in the present work was only 0.06%, and there were no large variations of the *T*_β_, as mentioned in Section 2.1, so that the volume fraction of the primary alpha phase remained at about 80%. The average size of the α phase of the three Ti-6Al-4V alloys was about 13 μm. That is, the adjustment of the oxygen content had a relatively limited refinement effect on the alpha phase under the same processing. In brief, the Ti-6Al-4V alloys with different oxygen contents used in this paper had similar microstructural characteristics.

**Figure 3:**
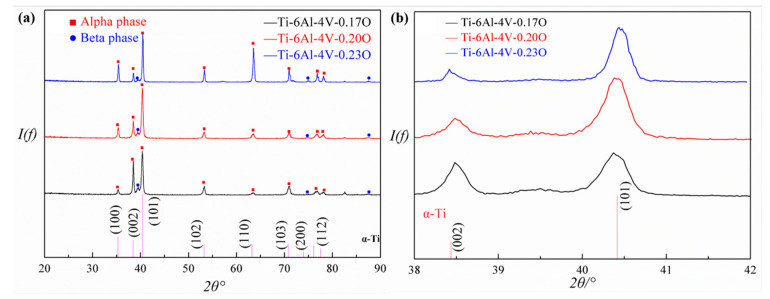
(**a**) XRD spectra and (**b**) *2θ* angle range from 38° to 42° of the Ti-6Al-4V alloy.

In addition to the volume fraction and the grain size of the primary alpha phase, the alpha phase lattice parameters are important characteristics as well. Figure 3a is the XRD spectra of the Ti-6Al-4V alloys with different oxygen contents. There were only two kinds of diffraction peaks in the XRD spectra, which stood for the alpha phase and the beta phase, respectively. Namely, there were no metal oxides’ precipitates in the present Ti-6Al-4V alloys, indicating the oxygen content in present Ti-6Al-4V alloys did not exceed the maximum solubility. Moreover, the intensity of the (101) diffraction peak increased slightly with the oxygen content. At the same time, the intensity of the (002) diffraction peak reduced obviously. A similar experimental result was found by Firstov et al. [22,23]. The reasonable explanation was that the orientation of the alpha phase in the Ti-6Al-4V alloys changed from (002) and (101) dominant jointly to (101) dominant alone with increasing oxygen content.

The XRD spectra within the *2θ* angle range from 38° to 42° of the Ti-6Al-4V alloys are shown in Figure 3b. There were two diffraction peaks, which represented the (002) and the (101) crystallographic planes, respectively, that appeared in such *2θ* angle range. It was obvious that both peaks in the three kinds of Ti-6Al-4V alloy shifted to left, which signified a decrease of the *2θ* angle. According to the Bragg equation, the lattice parameters could be obtained, as listed in Table 2. Apparently, both the lattice parameters of the alpha phase (*a* and *c*) and the ratio of *c* to *a* (*c/a*) increased with the increasing oxygen content, indicating that the oxygen atom occupied the octahedral interstices of the alpha phase [11], which would give rise to the lattice expansion. 

### 3.2. Effect of Oxygen Variation on the Micro-Mechanical Properties of Alpha Phase

**Figure 4:**
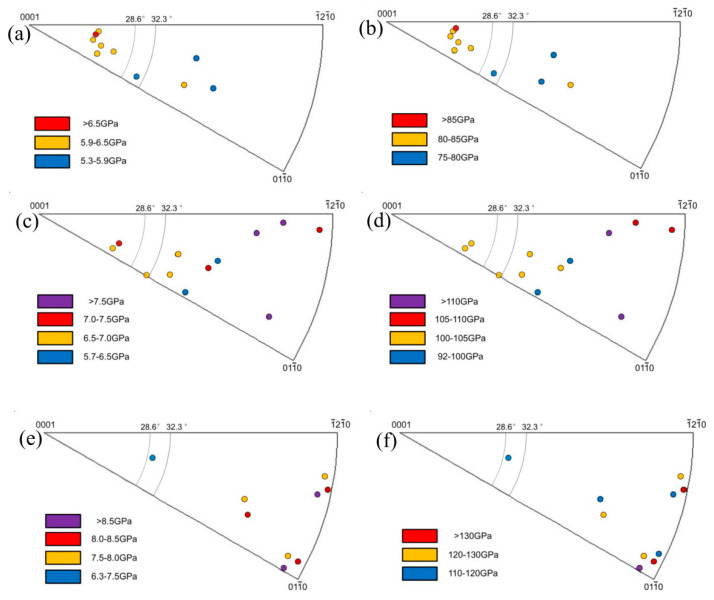
Hardness inverse pole figures (HIPFs) and modulus inverse pole figures (MIPFs) of (**a**,**b**) Ti-6Al-4V-0.17O, (**c**,**d**) Ti-6Al-4V-0.20O, and (**e**,**f**) Ti-6Al-4V-0.23O, respectively.

In order to better characterize the effect of oxygen content on the micro-mechanical properties of Ti-6Al-4V alloy, and then to provide useful information for analyzing fatigue behavior, nano-indentation experiments with alpha grains were systematically tested. The HIPF and MIPF of Ti-6Al-4V alloys with different oxygen contents are given in Figure 4.

Though there was dispersion, the average nano-hardness values of the Ti-6Al-4V alloys with different oxygen contents were 6.5 GPa, 7.5 GPa, and 8.5 GPa, respectively. Meanwhile, the values of average elasticity modulus of the three kinds of Ti-6Al-4V alloys were 85, 110, and 130, respectively, and are shown in Figure 4b,d,f. Both the elasticity modulus values and the nano-hardness values of the Ti-6Al-4V alloys increased with the oxygen content, which was in accord with the reference [24] and [25]. Moreover, the maximum hardness values of the Ti-6Al-4V-0.17O appeared at the grains orientated to the <0001> direction, which was in accord with the reference [26], as shown in Figure 4a. Moreover, the location of maximum hardness values varied from the <0001> direction to the $<\bar{1}2\bar{1}0>$ and $<01\bar{1}0>$ directions with the increasing oxygen content in Ti-6Al-4V alloys, as seen in Figure 4c,e. Churchman [27] found that the increment of the critical resolved shear stress (CRSS) varied in different slip systems, due to the variation of the oxygen contents in titanium alloys. The primary slip system was prismatic slip in the titanium alloys with lower oxygen content. However, the pyramidal slip would exceed the prismatic slip and become the dominating slip system when the oxygen content reached a critical level. The variation of oxygen content in titanium alloys would lead to the variation of the CRSS in different slip systems; thereby, the maximum hardness values varied with the increasing oxygen content. The theoretical angles between the indenter and the two pyramidal planes $\left\{11\bar{2}2\right\}$ and $\left\{10\bar{1}1\right\}$ were 28.6° and 32.3°, respectively, as shown in Figure 4 in stereograph. All of the minimum hardness values of the three grades of Ti-6Al-4V alloys appeared in the range 28.6° to 32.3°, which suggested that pyramidal slip was the dominating slip system. Welsch et al. [8] obtained similar results in Ti-6Al-4V alloy with 0.22% oxygen by weight percent. The elasticity modulus showed a similar variation tendency as the hardness, which is shown in Figure 4b,d,f, but with a smaller dispersion than the hardness values. Fizanne-Michel et al. [26] found similar results, that the grain orientation had little effect on the pure Ti with 0.25 wt.% oxygen content. Despite the oxygen content influencing markedly the CRSS, the elasticity modulus hardly changed with grain orientation. Thus, the elasticity modulus dispersed a little.

### 3.3. Effect of Oxygen Variation on Fatigue Properties

**Figure 5:**
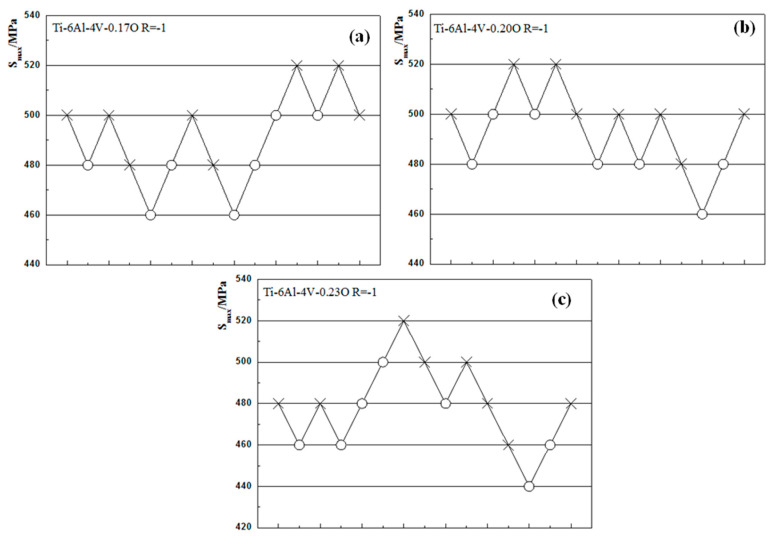
Experimental results of staircase method fatigue testing of Ti-6Al-4V alloys with different oxygen contents: (**a**) Ti-6Al-4V-0.17O, (**b**) Ti-6Al-4V-0.20O, (**c**) Ti-6Al-4V-0.23O. The circle symbol (○) represents that the specimen did not break and the cross symbol (×) represents that the specimen fractured after 10^7^ cycles of fatigue loading at this stress level.

The experimental results of the staircase method are shown in Figure 5. It can be seen from the figure that 15 specimens were employed during the test, and S_0_ = 460 MPa in Ti-6Al-4V-0.17O, S_0_ = 460 MPa in Ti-6Al-4V-0.20O, and S_0_ = 440 MPa in Ti-6Al-4V-0.23O, while the value of discretization range *d* was 20 MPa in all three kinds of Ti-6Al-4V alloy.

After performing high cycle fatigue tests on Ti-6Al-4V alloys with different oxygen contents, the measured fatigue strength is shown in Table 3. It can be seen that the fatigue strength changed slightly with the little variation of the oxygen contents, the fatigue performance of the Ti-6Al-4V-0.20O alloy was relatively better compared with the Ti-6Al-4V alloy with other oxygen contents, and the dispersion of high cycle fatigue life was the lowest.

**Figure 6:**
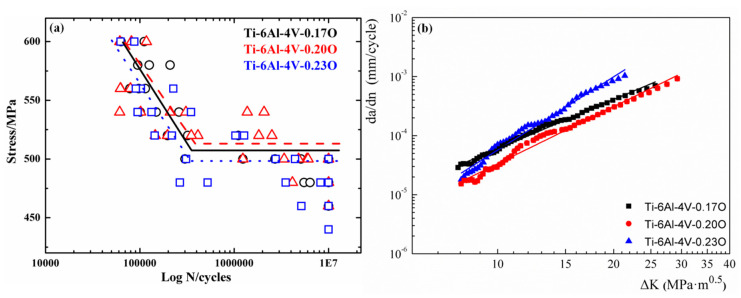
Fatigue properties of the Ti-6Al-4V alloys with different oxygen contents. (**a**) Stress-fatigue life curves, in which the black circle and line represent Ti-6Al-4V-0.17O alloy, the red triangle and dash represent Ti-6Al-4V-0.20O alloy, and the blue square and short dot represent Ti-6Al-4V-0.17O alloy. (**b**) Fatigue crack propagation rate curves.

The stress-fatigue life curves of the Ti-6Al-4V alloys with different oxygen contents are shown in Figure 6a. The power function empirical formula, which is called the S-N curve [28], was employed to match the data points in the single logarithmic coordinate system (S-logN), thus obtaining the aforementioned curves. The horizontal section of the curves represented the fatigue strength of each Ti-6Al-4V alloy, and the specific numerical values are listed in Table 3. It is shown in Figure 6a that the fatigue life of the three Ti-6Al-4V alloys was similar at upper stress levels (above 560 MPa). However, the Ti-6Al-4V-0.20O had the longest fatigue life when the stress level was below 560 MPa.

Figure 6b shows the fatigue crack propagation rate curves of the Ti-6Al-4V alloys with different oxygen contents, which could be well fitted to lines according to the Paris Formula. The fatigue crack propagation rate of the Ti-6Al-4V-0.20O alloy was the lowest among the three alloys investigated. Additionally, fatigue crack in the Ti-6Al-4V-0.17O alloy propagated faster than the Ti-6Al-4V-0.23O alloy at lower Δ*K* (≤10.5 MPa·m^1/2^) region. When the Δ*K* value surpassed 10.5 MPa·m^1/2^ the fatigue crack propagated fastest in Ti-6Al-4V-0.23O alloy. Smith and Piascik [29] found the elongated grain and the grain with specific crystal orientation would benefit fatigue crack propagation. To be more specific, when the *c*-axis of the alpha grains are parallel to the fatigue loading direction, that is, when the loading axis is perpendicular to the basal plane of the alpha grains, the cleavage surface of the basal plane or the near basal plane orientation is easily formed; and when the *c*-axis is vertical to the loading axis, the orientation of the grains is not conducive to the sliding of the cylinder surface and, thus, greatly hinders the expansion of the fatigue crack.

### 3.4. Characterization of Fatigue Fracture Morphologies

**Figure 7:**
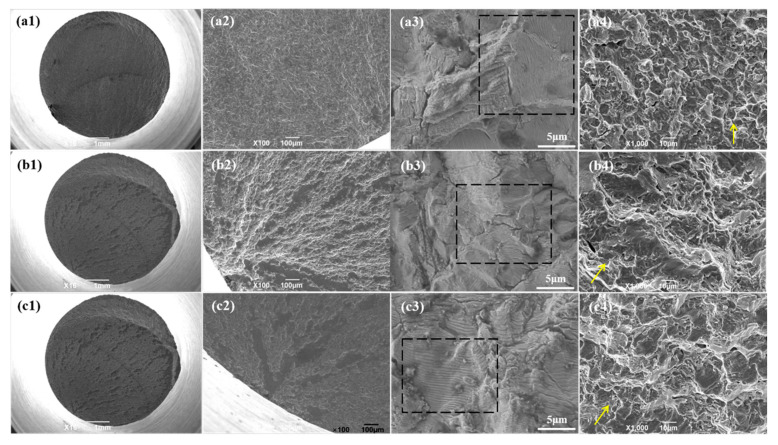
Fatigue fracture morphologies of the Ti-6Al-4V alloys with different oxygen contents (**a**) Ti-6Al-4V-0.17O, (**b**) Ti-6Al-4V-0.20O, and (**c**) Ti-6Al-4V-0.23O, (**1**) macroscopic fracture morphologies (**2**) fatigue source regions (**3**) fatigue propagation regions, (**4**) instantaneous fracture zones.

The fatigue fracture morphologies of the Ti-6Al-4V alloys with different oxygen contents are shown in Figure 7. As shown in Figure 7(a1,b1,c1), all of the fatigue fracture surfaces of the three Ti-6Al-4V alloys possessed three zones: The fatigue source region where the fatigue crack initiated, the fatigue propagation region where the fatigue crack propagated stably, and the instantaneous fracture region where the fatigue crack propagated dramatically until the specimen fractured. 

It can be seen from Figure 7(a1,b1,c1) that all the fatigue source regions of the three kinds of Ti-6Al-4V alloys located on the surface of the sample. Further magnifying the fatigue source regions, as shown in Figure 7(a2,b2,c2), it can be observed that there was no inclusion, fisheye morphology, or surface processing defects in this area. So, it was concluded that the reason why the fatigue source was formed on the surface of the Ti-6Al-4V alloy sample was neither due to processing defects nor materials defects, but because the stress distribution was large at the surface position and was small at the center position when the rotary bending load was applied; so the fatigue crack first sprouted on the surface of the sample. It can also be seen from the Figure 6 that the sections of the fatigue source regions were relatively flat. This is because the fatigue source regions were the earliest separated regions on the fatigue fracture. Under the continuous action of the cyclic load, the two separated sections continuously friction against each other and, finally, the area presented a relatively flat morphology.

Figure 7(a3,b3,c3) display the fatigue propagation regions of the three kinds of Ti-6Al-4V alloy, and there were striations in each alloy, which gave the evidence that the fatigue fracture mechanism was due to the dislocation slip. Theoretically, the number of striations was equal to the cycle index. However, firstly, the grain boundaries, the phase boundaries, and the secondary phase particles strongly inhibited the fatigue cracks. Secondly, subgrains produced by dislocation movement could disperse the stress concentration during the fatigue loading process, which generated a more homogeneous deformation. Thus, the fatigue striation characteristics vanished. Based on the above reasons, the number of actual observed striations was far less than the number of fatigue cycle index. 

The distance between the adjacent striations represented the fatigue crack extension after each cycle. The larger the distance, the faster the fatigue crack propagated. The average striation distance values were 0.4143 μm, 0.2449 μm, and 0.7067 μm, respectively in Ti-6Al-4V alloys with the increasing oxygen content, which indicated that fatigue cracks in the Ti-6Al-4V-0.23O alloy propagated fastest while the fatigue crack propagation rate was the minimum in the Ti-6Al-4V-0.20O alloy, which was well consistent with the fatigue crack propagation rate curves given in Figure 6b. Moreover, the fatigue cracks could be easily found at the fatigue propagation regions in the titanium alloys in Figure 7. The fatigue cracks mainly traversed the alpha grain in the two Ti-6Al-4V alloys with lower oxygen content, as seen in Figure 7(a3,b3). However, the fatigue cracks extended mainly along the phase and grain boundaries in Figure 7(c3).

The morphologies of the instantaneous fracture zones of the Ti-6Al-4V alloys with different oxygen contents are shown in Figure 7(a4,b4,c4). The fracture mode of the Ti-6Al-4V-0.17O alloy was a typical transgranular fracture mode, which is illustrated in Figure 7(a4). With the increasing oxygen content in the Ti-6Al-4V alloys, the fracture mode changed from the transgruanlar fracture mode to a mixture of intergranular fracture and transgruanlar fracture mode, as shown in Figure 7(b4). When the oxygen content reached 0.23% in titanium alloy, the fracture mode was the intergranular fracture, as shown in Figure 7(c4). 

The phenomenon of the change of fracture mode with the change of oxygen content can be attributed to the strong strengthening effect of oxygen on the alpha phase of titanium alloy. To be more specific, the fatigue cracks tended to propagate along the path of lower crack formation energy. With the increasing oxygen content in the titanium alloys, the alpha phase was strongly enhanced. Meanwhile, the degree of anisotropy of alpha grains increased, due to which the alpha/alpha grain boundaries and the alpha/beta phase boundaries became the main paths for the fatigue cracks’ propagation.

**Figure 8:**
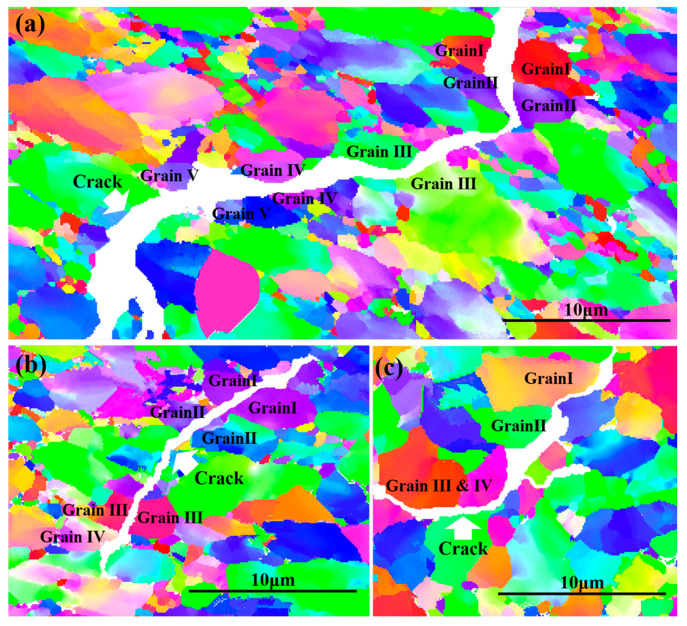
Electron Back-Scattered Diffraction (EBSD) micrographs of the fatigue crack propagation paths of (**a**) Ti-6Al-4V-0.17O, (**b**) Ti-6Al-4V-0.20O, and (**c**) Ti-6Al-4V-0.23O.

EBSD was employed to investigate the fatigue crack propagation paths in Ti-6Al-4V alloys with different oxygen content, and the micrographs are given in Figure 8. The fatigue cracks tended to propagate transgranularly in the Ti-6Al-4V-0.17O alloy. As shown in Figure 8a, the Grain I–V are the cracked grains. In the Ti-6Al-4V-0.20O alloy the fatigue cracks expanded both along the grain boundaries and inside the grain. As shown in Figure 8b, the Grain I–III are the cracked grains and the Grain IV is bypassed by cracks. However, the fatigue cracks extended prevailingly along the phase and grain boundaries in the Ti-6Al-4V-0.23O alloy. As shown in Figure 8c, the Grain I–IV are all bypassed by cracks.

The reason for this phenomenon is that, firstly, oxygen is one of the strongest alpha phase stabilizer elements of titanium alloy, and the alpha phase can be strongly enhanced by the increasing oxygen content [13]. Secondly, dislocation pileup and cyclic substructure will be formed in the beta phase, which will intensify the beta phase during cyclic loading [30]. Meanwhile, the alpha phase shows an obvious cyclic softening [31]. Thirdly, the alpha phase is of hexagonal close-packed structure; the increasing oxygen content will increase the degree of anisotropy. The aforementioned factors will strongly influence the strength of the alpha and beta phases in different degrees. Moreover, the overlaid effects will be reflected straightly on the fatigues’ crack propagation paths. Therefore, the crack extended transgranularly in Ti-6Al-4V-0.17O and Ti-6Al-4V-0.20O, whereas the crack propagated intergranularly in Ti-6Al-4V-0.23O.

### 3.5. Dislocation Structure Features of the Ti-6Al-4V Alloy after Fatigue Loading 

**Figure 9:**
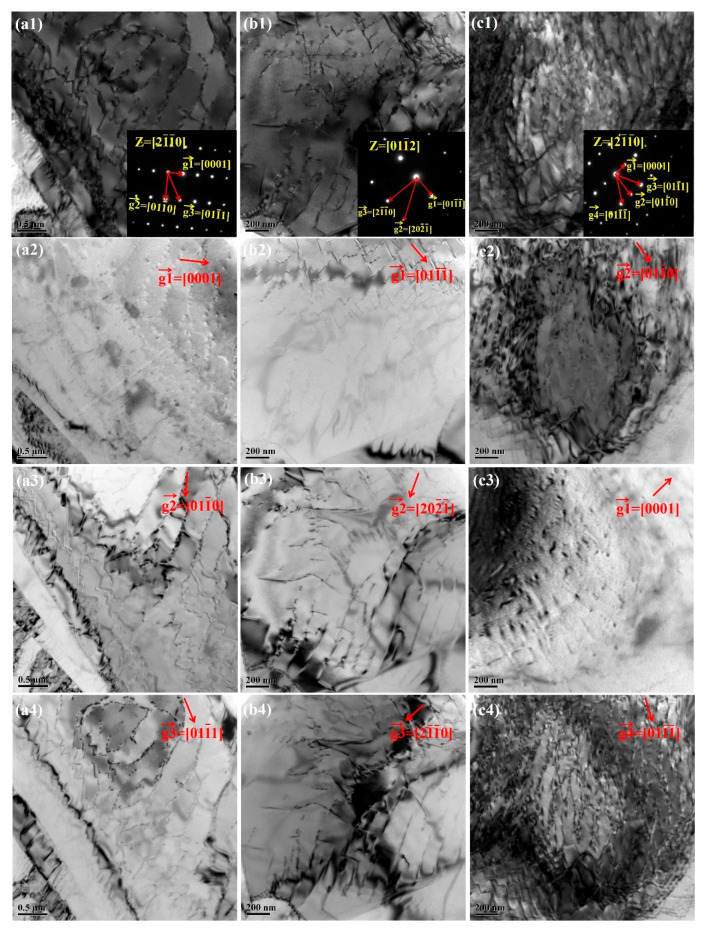
Micrographs of various dislocations in (**a**) Ti-6Al-4V-0.17O alloy, (**b**) Ti-6Al-4V-0.20O alloy, (**c**) Ti-6Al-4V-0.23O alloy, (**a1**) Z = [2$\bar{1}\bar{1}$0], (**a2**) $\vec{g}$_1_ = [0001], (**a3**) $\vec{g}$_2_ = [01$\bar{1}0$], (**a4**) $\vec{g}$_3_ = [01$\bar{1}$1], (**b1**) Z = [01$\bar{1}$2], (**b2**) $\vec{g}$_1_ = [01$\bar{1}\bar{1}$], (**b3**) $\vec{g}$_2_ = [20$\bar{2}\bar{1}$], (**b4**) $\vec{g}$_3_ = [2$\bar{1}\bar{1}$0], (**c1**) Z = [2$\bar{1}\bar{1}$0], (**c2**) $\vec{g}$_2_ = [01$\bar{1}$0], (**c3**) $\vec{g}$_1_ = [0001], (**c4**) $\vec{g}$_4_ =[01$\bar{1}\bar{1}$].

The micrographs of dislocations in three Ti-6Al-4V alloys with different oxygen contents investigated by TEM are illustrated in the Figure 9. It was obvious that the dislocations were corrugated. Moreover, the dislocations fluctuated more violently and distributed more inhomogeneously with the increasing oxygen content in the titanium alloys. The reasons for the above phenomenon were as follows: Firstly, the lattice constant of the alpha phase increased with the oxygen content, which led to the increasing degree of anisotropy. Thus, the differences in activation energy between different slip systems intensified significantly with the increasing oxygen content. Consequently, dislocation motion was strongly hindered by grain, phase, and subgrain boundaries in Ti-6Al-4V alloy with higher oxygen content, which led to increasing degree of inhomogeneity. Secondly, there was strong elastic interaction between the oxygen atoms occupying the octahedral interstices and the dislocations, which increased the dislocation slip resistance. Concomitantly, dislocation mobility decreased and dislocations accumulated more, resulting in more obvious dislocation pileups with increasing oxygen content. M. S. Pham et al. [30] found that both solid solution and interstitial atom had strong influence on the characteristics of dislocations in steel with a fairly high oxygen content. Especially, they found that the dislocation lines tended more intensely to form short-range ordered structure at the place with dense atoms. Such structure caused increasingly uneven distribution of stress at dislocation lines and the final outcome was the corrugated dislocation lines. The oxygen atom could pin the dislocation lines; thus, the dislocation lines were separated in several segments. Each segment continued the motion, despite the pinned portion, giving further rise to the formation of the corrugated dislocation lines. The possibility for the dislocations to be pinned increased with the oxygen content. Hence, the dislocations fluctuated more violently, as seen in Figure 9c.

The double-beam diffraction mode was used to verify the dislocations types of Ti-6Al-4V alloys with different oxygen contents, and the micrographs are also shown in Figure 9. In the Ti-6Al-4V-0.17O shown in Figure 9a, the beam direction was B = $\left[2\bar{1}\bar{1}0\right]$. Thus, the $<\vec{a}>$ dislocations were out of contrast in Figure 9(a2) and the $<\vec{c}>$ dislocations were out of contrast in Figure 9(a3). Similarly, in Ti-6Al-4V-0.20O the $<\vec{c}+\vec{a}>$ dislocations were out of contrast in Figure 9(b2) and the $<\vec{c}>$ dislocations were out of contrast in Figure 9(b4). In Ti-6Al-4V-0.23O the $<\vec{a}>$ dislocations were out of contrast in Figure 9(c2), the $<\vec{c}>$ dislocations were out of contrast in Figure 9(c3), and $<\vec{c}+\vec{a}>$ dislocations were out of contrast in Figure 9(c4). It is obvious in Figure 9 that the number of $<\vec{c}+\vec{a}>$ dislocations increased with the oxygen content. Zaefferer [32] proved that the interstitial atoms strongly impede the basal slip system and the prismatic slip system. However, the interstitial atoms contribute less to hindering the pyramidal slip system. Thus, the increasing number of $<\vec{c}+\vec{a}>$ dislocations can be ascribed to the activation of the prismatic slip systems.

## 4. Conclusions

The effects of oxygen variation on the high cycle fatigue behavior of Ti-6Al-4V were investigated in this paper. The following main conclusions can be drawn:

- The volume fraction of primary alpha phase and the alpha-Ti lattice constants (*a*, *c*) and the *c/a* ratio increased a little with increasing oxygen content. The microstructure characteristics (alpha phase volume fraction and grain size) of the Ti-6Al-4V alloy were not significantly changed by the fine adjustment of the oxygen content.
- The values of elasticity modulus and nano-hardness increased with the oxygen content in Ti-6Al-4V alloys. The HIPFs and MIPFs illustrated that the variation of oxygen content in Ti-6Al-4V alloys would lead to the variation of the CRSS in different slip system. Such results aligned well with the fact that the pyramidal slip exceeded the prismatic slip and became the dominating slip system when the oxygen content increased in Ti-6Al-4V alloys.
- By controlling the oxygen content in the Ti-6Al-4V alloy, both the fatigue performance and the fatigue performance scatter of the Ti-6Al-4V alloy can be controlled. The fatigue performance of the Ti-6Al-4V-0.20O alloy was relatively better, compared with the Ti-6Al-4V alloy with other oxygen contents, and the dispersion of high cycle fatigue life was the lowest. Fatigue cracks propagated transgranularly in both Ti-6Al-4V-017O and Ti-6Al-4V-0.20O alloys, and the cracks tended to extend intergranularly in the Ti-6Al-4V-0.23O alloy. Such phenomenon indicated that the fatigue fracture micro-mechanisms varied from transcrystalline fracture to intercrystalline fracture.
- The dislocations were corrugated and fluctuated more intensely with the increasing oxygen content. The number of $<\vec{c}+\vec{a}>$ dislocations increased obviously with increasing oxygen content, which suggested that the number of the activated prismatic slip systems increased in Ti-6Al-4V alloys.

## Figures and Tables

**Table 1:** Chemical compositions of the present Ti-6Al-4V alloys by weight percent.

Alloy	Ti	Al	V	O	Fe	C	N	Si	H
Ti-6Al-4V-0.17O	Bal.	6.19	4.13	0.17	0.17	0.008	0.011	0.017	<0.001
Ti-6Al-4V-0.20O	Bal.	6.36	4.19	0.20	0.18	0.008	0.013	0.014	<0.001
Ti-6Al-4V-0.23O	Bal.	6.22	4.11	0.23	0.18	0012	0.013	0.013	<0.001

**Table 2:** The lattice parameters of the Ti-6Al-4V alloy.

Alloy	*a*/nm	*c*/nm	*c*/*a*
Ti-6Al-4V-0.17O	0.2944	0.4677	1.58865
Ti-6Al-4V-0.20O	0.2945	0.4679	1.58879
Ti-6Al-4V-0.23O	0.2948	0.4685	1.58921

**Table 3:** Fatigue properties of the Ti-6Al-4V alloy with different oxygen contents.

Alloy	Fatigue Limit $\hat{\mu }$/MPa	K of the Inclined Portion S-N Curves	Standard Deviation $\hat{\delta }$	90% Percentiles of the Fatigue Limit Distribution
Ti-6Al-4V-0.17O	510.00	9.396	19.45	480
Ti-6Al-4V-0.20O	512.86	10.887	14.16	480
Ti-6Al-4V-0.23O	498.57	9.715	27.39	468
