# Correction: Tang, L. et al., Effect of Oxygen Variation on High Cycle Fatigue Behavior of Ti-6Al-4V Titanium Alloy. *Materials* 2020, *13*, 3858

**DOI:** 10.3390/ma13235364

**Published:** 2020-11-26

**Authors:** Luyao Tang, Jiangkun Fan, Hongchao Kou, Bin Tang, Jinshan Li

**Affiliations:** 1 State Key Laboratory of Solidification Processing, Northwestern Polytechnical University, Xi’an 710072, Shaanxi, China; christinatang@mail.nwpu.edu.cn (L.T.); hchkou@nwpu.edu.cn (H.K.); toby@nwpu.edu.cn (B.T.); 2 National & Local Joint Engineering Research Center for Precision Thermoforming Technology of Advanced Metal Materials, Xi’an 710072, Shaanxi, China

The author wishes to make the following correction to this paper [1]. After careful comparison and examination, we found that Figure 7(b1) and Figure 7(c1) are the same. The reason for the error is that this manuscript underwent many revisions during the writing and submission process. As such, Figure 7(c1) was wrongly copied twice accidentally in the combined editing of Figure 7. Due to the duplication of Figure 7(c1), please replace:

**Figure 7:**
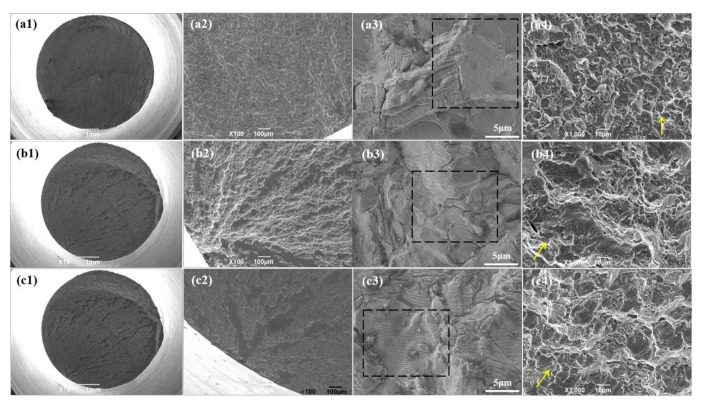
Fatigue fracture morphologies of the Ti-6Al-4V alloys with different oxygen contents: (**a**) Ti-6Al-4V-0.17O, (**b**) Ti-6Al-4V-0.20O, and (**c**) Ti-6Al-4V-0.23O, (**1**) macroscopic fracture morphologies (**2**) fatigue source regions, (**3**) fatigue propagation regions, and (**4**) instantaneous fracture zones.

with the following:

**Figure 7:**
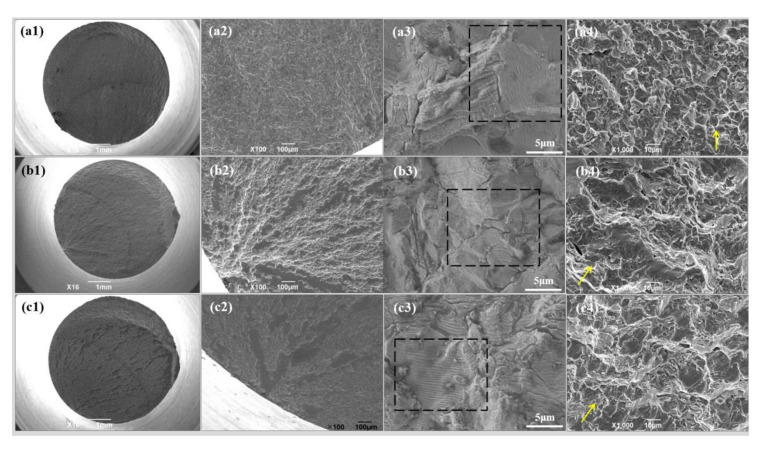
Fatigue fracture morphologies of the Ti-6Al-4V alloys with different oxygen contents: (**a**) Ti-6Al-4V-0.17O, (**b**) Ti-6Al-4V-0.20O, and (**c**) Ti-6Al-4V-0.23O, (**1**) macroscopic fracture morphologies, (**2**) fatigue source regions, (**3**) fatigue propagation regions, and (**4**) instantaneous fracture zones.

The correction of Figure 7 will not influence the original analysis of the results or the conclusion of this paper. The authors would like to apologize for any inconvenience caused to the readers by these changes.
